# Exploring the Link Between Somatic and Psychiatric Symptoms in Patients With Medically Unexplained Physical Symptoms: A Cross-Sectional Survey From North India

**DOI:** 10.7759/cureus.65984

**Published:** 2024-08-02

**Authors:** Abhivandana Pallati, Amandeep Singh, Piyush Ranjan, Nandini Rawat, Siddharth Sarkar, Gaurishanker Kaloiya, Upendra Baitha, Ashish D Upadhyay, Bindu Prakash, Ranveer S Jadon

**Affiliations:** 1 Medicine, All India Institute of Medical Sciences, New Delhi, New Delhi, IND; 2 Psychiatry, All India Institute of Medical Sciences, New Delhi, New Delhi, IND; 3 Clinical Psychology, All India Institute of Medical Sciences, New Delhi, New Delhi, IND; 4 Biostatistics, All India Institute of Medical Sciences, New Delhi, New Delhi, IND

**Keywords:** stressful life events, cross-sectional survey, somatic symptoms severity, psychiatric comorbidities, medically unexplained physical symptoms

## Abstract

Background: The association between somatic symptoms and psychiatric co-morbidities remains unexplored among patients with medically unexplained physical symptoms (MUPS) in Asian populations. This study aims to bridge this gap by investigating psychiatric morbidities and their determinants among patients presenting with MUPS in an Indian setup.

Methodology: This cross-sectional study, conducted in the outpatient department (OPD) of a tertiary care hospital in India, assessed 200 patients diagnosed with MUPS. Assessment tools, such as the Somatic Symptom Scale (SSS-8), Presumptive Stressful Life Event Scale (PSLES), and Depression, Anxiety, and Stress Scale (DASS), were administered to collect data.

Results: The study examined patients (mean age 36.51±9.82 years), predominantly comprising females (67.5%), presenting with MUPS. Common presenting symptoms were general (96.3%), musculoskeletal pain (91.7%), and gastrointestinal symptoms reported by 81.7%. Medium somatic symptom severity (57%) was more prevalent in females. Prevalent psychiatric co-morbid conditions included depression (mild: 22.0%, moderate: 26.5%), moderate anxiety (41.5%), and moderate stress (26%). Strong associations were observed between the SSS-8 score and depression (χ²(6, N = 200) = 49.26, p < 0.001), anxiety (χ²(8, N = 200) = 37.90, p < 0.001), stress (χ²(6, N = 200) = 44.45, p < 0.001), and the experience of stressful life events (χ²(3, N = 200) = 6.5, p < 0.05).

Conclusion: The study indicates an intertwined association between MUPS and psychiatric disorders. Individuals with MUPS commonly experience heightened anxiety and depression, emphasizing the complex interplay between somatic symptoms and emotional well-being. Consideration of environmental and social factors may be crucial for a comprehensive understanding.

## Introduction

Medically unexplained physical symptoms (MUPS) pose a significant healthcare challenge, marked by somatic complaints persisting without a definitive medical diagnosis despite exhaustive evaluations [1]. This diagnostic uncertainty often leaves patients without clear treatment plans, fostering frustration and distress, thereby exacerbating both somatic and psychological distress and significantly affecting overall well-being [2,3].

The intricate interplay between stressors, somatic symptoms, and psychiatric comorbidities highlights the multifaceted nature of MUPS [4], often resulting in heightened anxiety, depression, and functional impairment [5]. Traumatic life events, such as childhood adversity and interpersonal conflicts, are pivotal in both the onset and perpetuation of MUPS [6], contributing to its complexity. Studies worldwide have explored the association between somatic complaints and psychological comorbidities among MUPS patients. Barsky and Borus elucidated a bidirectional relationship between somatic symptoms and psychiatric disorders, indicating how psychological distress can manifest as physical symptoms and vice versa [7]. Similarly, Kroenke et al. demonstrated high rates of depression, anxiety, and stress among individuals with MUPS, suggesting a significant overlap between somatic and psychological domains [8].

These findings emphasize the significance of addressing both physical and psychological aspects in MUPS management for holistic care. However, it is vital to recognize that the severity and expression of somatic symptoms and related psychological factors are shaped by the socio-cultural context of each population. Thus, adopting a tailored approach rather than a one-size-fits-all strategy is imperative to understand MUPS [9].

Despite the significant occurrence of MUPS among Asian outpatient department (OPD) attendees, research into the severity of somatic symptoms and psychiatric comorbidities in Asian countries remains limited [10,11]. The burden of managing MUPS further emphasizes the necessity of addressing both its physical and psychological dimensions for comprehensive care [12,13]. Therefore, our study aims to bridge this gap by investigating the association between somatic symptoms, psychiatric morbidities, and psychological characteristics of individuals diagnosed with MUPS in an Indian setup.

## Materials and methods

Study design

This cross-sectional study was conducted in the Medicine OPD of a tertiary care hospital in North India. Ethical approval was obtained from the Institution Ethics Committee (IEC PG-715/25.11.2021, OT-02/24.03.2022). 

Study participants

Two hundred patients visiting the Medicine OPD were evaluated for MUPS following specific inclusion and exclusion criteria. Inclusion criteria comprised patients diagnosed with MUPS, aged 18-60 years with no gender-based exclusion, and were willing to participate. MUPS were characterized as physical symptoms that lack an identifiable organic cause despite rigorous medical evaluation. Exclusion criteria included individuals with neurological or severe medical conditions hindering participation, previously diagnosed psychiatric disorders, and those with sufficient medical explanations for symptoms. Purposive sampling was used, with patients referred by clinicians after a comprehensive examination and evaluation. 

Survey questionnaire

A proforma designed for this study recorded sociodemographic data, including age, gender, marital status, educational level, employment status, and occupation. The following scales were administered to assess psychiatric comorbidities and the psychological characteristics of the patients:

Somatic Symptom Scale-8 (SSS-8)

The SSS-8 includes eight items that measure the severity and impact of somatic symptoms over the past seven days. The SSS-8 exhibits good reliability (Cronbach's α = 0.81), demonstrates high content validity from the PHQ-15, positive associations with depression and anxiety, and a stable factor structure across demographics, establishing it as a concise and valid tool for assessing somatic symptoms [14].

Depression Anxiety Stress Scale (DASS-21)

The DASS-21 is a self-report questionnaire designed to measure levels of depression, anxiety, and stress. It demonstrates good reliability and validity, with Cronbach's α coefficients ranging from 0.88 to 0.94. The DASS-21 is widely used in research and clinical settings to assess three negative emotional states accurately [15].

Presumptive Stressful Life Events Scale (PSLES)

The PSLES is a reliable measure designed to assess stressful life events experienced by individuals, adapted from the Holmes and Rahe Social Readjustment Rating Schedule. With 51 defined life events, it has been reconstructed and standardized for the Indian population, demonstrating high reliability (0.87) and validity in studies related to both mental and physical health [16]. This comprehensive toolkit facilitated a thorough assessment of the psychological and psychiatric comorbidities of the patients.

Data collection

Patients diagnosed with MUPS were directed by clinicians in the OPD to a designated room for systematic data collection, ensuring patient privacy. Trained medical professionals conducted the process, briefing patients about the purpose of the study, and obtaining written consent. This structured approach upheld ethical standards and facilitated comprehensive data collection while prioritizing patient confidentiality.

Statistical analysis

Categorical variables were presented as absolute and relative frequencies, and continuous variables as mean with standard deviation. The association between variables was assessed using chi-square and Fisher exact tests. The statistical analysis aimed to uncover relationships and patterns within the collected data, providing insights into the interplay of psychiatric comorbidities among individuals diagnosed with MUPS. The analysis was considered statistically significant at p < 0.05.

## Results

Socio-demographic and medical characteristics of the patients

The socio-demographic profile of patients diagnosed with MUPS in the OPD of medicine is delineated in Table 1. The average age of patients was 36.51±9.82 years, demonstrating a notable female preponderance, constituting approximately 68% of the total sample. Regarding socioeconomic status (SES), the majority came from lower-middle-class households (42%), followed by 37.5% from upper-lower-class backgrounds (Table 1).

**Table 1:** Socio-demographic characteristics of the patients (N=200)

Socio-demographic characteristics	n (%)
Age (36.51±9.82 years)	
<20 years	7 (3.5)
20-40 years	113 (56.5)
40-60 years	80 (40)
Gender	
Female	135 (67.5)
Male	65 (32.5)
Marital status	
Married	159 (79.5)
Unmarried	40 (20)
Widowed	1 (0.5)
Socioeconomic status	
Lower class	6 (3)
Upper lower class	75 (37.5)
Lower middle class	84 (42)
Upper middle class	34 (17)
Upper class	1 (0.5)

Among patients presenting with MUPS, 96.3% reported experiencing general somatic complaints, with musculoskeletal tension symptoms emerging as the predominant manifestation reported by 92% of the patients. Furthermore, 81.7% reported gastrointestinal symptoms, while headaches also emerged prevalent concern, reported by 89% of the patients (Table 2).

**Table 2:** Common presenting symptoms of the patients (N=200) GI = Gastrointestinal

Common presenting symptoms	Frequency (%)
Cardiopulmonary symptoms (palpitations, discomfort, breathlessness, sweating)	56
GI Symptoms (abdominal pains, feeling bloated, diarrhea, nausea)	81.7
Musculoskeletal symptoms (pains, joint pain, weakness, backache, numbness or tingling sensations)	91.7
General symptoms (concentration difficulties, fatigue)	96.3
Headaches	89

Association of psychological characteristics with socio-demographic characteristics

The relationship between the psychological parameters of patients diagnosed with MUPS and their corresponding sociodemographic characteristics is illustrated in Table 3. This table presents a comprehensive overview of how various psychological factors, such as depression, anxiety, and stress levels, are influenced by demographic variables including age, gender, and SES among patients diagnosed with MUPS. 

**Table 3:** Association of psychological parameters with socio-demographic characteristics SSS-8 = Somatic Symptom Scale - 8, DASS-21 = Depression, Anxiety, and Stress Scale, PSLES - Presumptive Stressful Life Event Scale p = 0.001* = statistically significant at p<0.001, as determined by chi-square test. p = 0.011* = statistically significant at p<0.01, as determined by chi-square test. p < 0.05*= statistically significant at p<0.05, as determined by chi-square test.

Scales	Socio-demographic characteristics (N=200)							
	Age (N=200) (Mean±SD)	Gender, n (%)		SES, n (%)				
		Male (n=65)	Female (n=135)	Lower (n=6)	Upper lower (n=75)	Lower middle (n=84)	Upper middle (n=34)	Upper (n=1)
SSS-8								
Low (n=31)	35.03±9.6	24 (36)	7 (6)	1 (16)	6 (8)	17 (20)	7 (21)	0 (0)
Medium (n=103)	35.93±9.5	26 (40)	77 (57)	5 (84)	45 (60)	37 (44)	15 (44)	1 (100)
High/very high (n=66)	38.0±10.2	15 (24)	51 (37)	0 (0)	24 (32)	30 (36)	12 (35)	0 (0)
P-value	p=0.27	p=0.001*		p=0.116				
DASS-21								
Depression								
Normal (n=92)	36.2±10.0	38 (58)	54 (40)	3 (50)	30 (40)	43 (51.2)	15 (44.1)	1 (100)
Mild (n= 44)	34.3±9.9	17 (26)	30 (22)	0 (0)	18 (24)	16 (19)	10 (29.4)	0 (0)
Moderate (n=53)	37.5±8.6	9 (14.5)	43 (31)	2 (33.3)	20 (26.7)	23 (27.4)	8 (23.5)	0 (0)
Severe (n=11)	41.03±11.3	1 (1.5)	10 (7)	1 (16.7)	7 (9.3)	2 (2.4)	1 (2.9)	0 (0)
Extremely severe (n= 0)	0 (0)	0 (0)	0 (0)	0 (0)	0 (0)	0 (0)	0 (0)	0 (0)
P-value	p =0.760	p=0.011*		p=0.50				
Anxiety								
Normal (n=37)	36.3±9.0	16 (24.6)	21 (15.6)	1 (16.7)	14 (18.7)	14 (16.7)	8 (23.5)	0 (0)
Mild (n=58)	36.4±9.5	18 (27.7)	40 (29.6)	2 (33.3)	24 (32)	25 (29.8)	6 (17.6)	1 (100)
Moderate (n=83)	36.1±10.4	23 (35.4)	60 (44.4)	0 (0)	30 (40)	37 (44)	16 (47.1)	0 (0)
Severe (n=16)	38.6±9.4	6 (9.2)	10 (7.4)	3 (50)	4 (5.3)	6 (7.1)	3 (8.8)	0 (0)
Extremely severe (n=6)	39.2±9.4	2 (3.1)	4 (3)	0 (0)	3 (4)	2 (2.4)	1 (2.9)	0 (0)
P-value	p=0.88	p=0.53		p=0.20				
Stress								
Normal (n=84)	35.03±9.1	24 (36.9)	60 (44.4)	1 (16.7)	29 (38.7)	35 (41.7)	18 (52.9)	1 (100)
Mild (n=57)	36.03±10.9	24 (36.9)	33 (24.4)	1 (16.7)	23 (30.7)	24 (28.6)	6 (17.6)	0 (0)
Moderate (n=52)	36.03±9.5	14 (21.5)	38 (28.1)	0 (0)	18 (24)	25 (29.8)	9 (26.5)	0 (0)
Severe (n=7)	38.03±11.4	3 (4.6)	4 (3)	4 (66.7)	5 (6.7)	0 (0)	1 (2.9)	0 (0)
Extremely severe (n=0)	0	0 (0)	0 (0)	0 (0)	0 (0)	0 (0)	0 (0)	0 (0)
P-value	p=0.765	p=0.25		p<0.05*				
PSLES								
Yes events (n=83)	36.23±9.0	24 (37)	59 (44)	2 (67)	33 (44)	34 (40)	14 (41)	0 (0)
No events (n=117)	35.83±9.7	41 (63)	76 (56)	4 (33)	42 (56)	50 (60)	20 (59)	1 (100)
P-value	p=0.76	p=0.362		p=0.8567				

Prevalence of Somatic Symptom Severity in the Patients

Among 200 patients, 15.5% (n=31) exhibited low symptom severity (scores ranging from 4 to 7), 51.5% (n=103) reported medium symptom severity (scores ranging from 8 to 11), and 33% (n=66) demonstrated high and very high symptom severity (scores ranging from 12 to 15) based on the SSS-8 scoring criteria. Significant gender disparities were observed, with females displaying markedly elevated levels of somatic symptom severity compared to males, χ²(2, N = 200) = 33.58, p < 0.001. Females predominated in the medium and high severity categories relative to males. On the other hand, no statistically significant difference was observed across SES, χ²(8, N = 200) = 8.23, p = 0.116.

Prevalence of Depression, Anxiety, and Stress levels in the patients

The incidence of depression is notably higher among females compared to males, χ²(3, N = 200) = 11.80, p < 0.01. This disparity is particularly pronounced in females, especially in the moderate and severe categories. Analysis of depression levels indicates no substantial differences across SES, χ²(12, N = 200) = 10.43, p = 0.50.

Although the prevalence of anxiety is elevated in females across all levels, the statistical analysis yields no significant difference, χ²(4, N = 200) = 11.8, p = 0.532, indicating that the observed difference lacks statistical significance. Analysis of anxiety levels across SES categories reveals no statistically significant differences, χ²(16, N = 200) = 22.3, p = 0.203.

While assessing stress, there was no statistically significant association between gender and stress levels, χ²(3, N = 200) = 4.06, p = 0.25. Although females tended to experience higher stress levels, particularly in the mild and moderate categories, this disparity lacked statistical significance. An important observation pertains to the association between stress levels and SES, χ²(12, N = 200) = 46.77, p < 0.05. Among SES categories, approximately 30% (n=25) experience moderate stress among the lower-middle-class group, followed closely by upper-lower-class individuals (24%; n=18). Stress severity predominantly presents within the lower-middle class, particularly in the normal and mild categories.

Experience of Stressful Life Events by the Patients

Among the 200 patients included in the study, approximately 42% (n=83) reported experiencing a significant stressful life event at some point in their lives. Among these individuals, females reported more such experiences, comprising 44% (n=59) of the subgroup. Conversely, the majority of patients, constituting 58% (n=117), did not report experiencing any significant stressful life events throughout their lifetime. Analysis revealed no significant statistical differences across gender, χ²(1, N = 200) = 0.83, p = 0.362, and SES, χ²(4, N = 200) = 1.1, p = 0.856.

Association of somatic symptom severity scores with depression, anxiety, and stress levels in patients with MUPS

Table 4 presents the relationship between SSS-8 and levels of depression, anxiety, and stress among 200 participants. 

**Table 4:** Association between somatic symptom severity and depression, anxiety, and stress levels p<0.001*= statistically significant at p<0.001, as determined by chi-square test.

Variables	SSS-8 (N=200), n (%)			
	Low (n=31)	Medium (n=103)	High & very high (n=66)	P-value
					Depression				
Normal (n=92)	26 (83.9)	56 (54.4)	10 (15.2)	<0.001*
Mild (n=44)	4 (12.9)	22 (21.4)	18 (27.3)	
Moderate (n=53)	1 (3.2)	21 (20.4)	31 (47)	
Severe (n=11)	0 (0)	4 (3.9)	7 (10.6)	
Extremely severe (n=0)	0 (0)	0 (0)	0 (0)	
Anxiety				
Normal (n=37)	14 (45.2)	21 (20.4)	2 (3)	<0.001*
Mild (n=58)	9 (29)	35 (34)	14 (21.2)	
Moderate (n=83)	8 (25.8)	39 (37.9)	36 (54.5)	
Severe (n=16)	0 (0)	6 (5.8)	10 (15.2)	
Extremely Severe (n=6)	0 (0)	2 (1.9)	4 (6.1)	
Stress				
Normal (n=84)	20 (64.5)	56 (54.4)	8 (12.1)	<0.001*
Mild (n=57)	9 (29)	26 (25.2)	22 (33.3)	
Moderate (n=52)	2 (6.5)	18 (17.5)	32 (48.5)	
Severe (n=7)	0 (0%)	3 (2.9)	4 (6.1)	
Extremely severe (n=0)	0 (0)	0 (0)	0 (0)	

Elevated SSS-8 scores were significantly associated with higher levels of depression, χ²(6, N = 200) = 49.26, p < 0.001. Notably, 47% (n=31) of patients with high and very high symptom severity also reported moderate levels of depression. A similar trend was observed for anxiety, with increasing SSS-8 scores correlating with elevated anxiety levels, χ²(8, N = 200) = 37.90, p < 0.001. Approximately 55% (n=36) of patients with high and very high symptom severity experienced moderate levels of anxiety. Similarly, higher SSS-8 scores were linked to heightened stress levels, χ²(6, N = 200) = 44.45, p < 0.001, where approximately 49% (n=32) of patients with high and very high symptom severity reported moderate stress levels.

Association of somatic symptom severity scores with stressful live events in patients with MUPS

The association between SSS-8 and life events measured by the PSLES among 200 participants has been presented in Table 5. Individuals experiencing high somatic symptom severity (12-15), and very high severity (16-32) reported more traumatic life events compared to those with low (4-7) or medium (8-11) severity. A statistically significant association was observed between somatic symptom severity and the occurrence of life events, χ²(3, N = 200) = 6.5, p < 0.05.

**Table 5:** Association between somatic symptom severity (SSS-8) and life events measured by the presumptive stressful life events scale (PSLES) p<0.025*= statistically significant at p<0.05, as determined by chi-square test.

PSLES	SSS-8 (N=200)				P-value
	Low (4-7)	Medium (8-11)	High (12-15)	Very high (16-32)	
Yes events (n=82)	7	42	31	2	<0.025*
No events (n=118)	24	61	33	0	

## Discussion

MUPS poses a significant challenge in healthcare due to its elusive nature and its burden on patients and healthcare systems [10,17]. Our study sheds light on the association between MUPS and various psychiatric comorbidities and psychological characteristics, as assessed by the SSS-8, DASS, and stressful life events experienced by the patients.

The association between high SSS-8 scores and elevated depression, anxiety, and stress, as evident in our results (p < 0.001), aligns with previous research as well. Elevated scores on the DASS suggest the presence of comorbid psychiatric conditions like depression and anxiety, which can exacerbate or be a consequence of MUPS. The psychological basis for this relationship is that mental health issues can manifest as physical symptoms when no medical explanation is evident. Conversely, the chronic stress and frustration of dealing with persistent and unexplained symptoms can lead to or worsen psychiatric conditions. This cycle can create a feedback loop where psychological and physical symptoms reinforce and sustain each other [18,19]. The bidirectional relationship between MUPS and psychiatric conditions signifies that MUPS may act as both a manifestation and precursor of psychological distress. Recent research supports this bidirectional understanding, suggesting that individuals with psychiatric disorders may be more susceptible to developing MUPS. At the same time, the distress caused by unexplained symptoms can contribute to the onset of psychiatric conditions [7,20].

While it is true that there is evidence suggesting a correlation between somatic symptoms and psychological distress, it is important to consider other factors that may contribute to this association. Some studies have suggested that the relationship between somatic symptoms and psychological distress may be more complex than previously thought [21]. For instance, a study proposed that the association between somatic symptoms and psychological distress may be influenced by individual differences in the cognitive and emotional processing of physical sensations [22]. Additionally, the presence of comorbid psychiatric conditions such as depression and anxiety does not necessarily indicate a causative relationship with MUPS. These conditions may co-occur with MUPS without directly influencing its onset or exacerbation [23].

Previous research has underscored the connection between life events and the emergence of somatic symptoms. This association suggests that stressful life events can precipitate physical symptoms through psychological mechanisms [6]. The psychological basis lies in the stress-response system, where heightened stress levels triggered by life events may lead to somatic symptom expression [24]. Additionally, cognitive and emotional processes play a role, as individuals may interpret and respond to stressors in ways that manifest as physical ailments [25]. Stressful life events, measured by the PSLES, demonstrated a statistically significant association with high SSS-8 scores, underscoring the role of environmental stressors in contributing to MUPS.

Furthermore, while chronic stress and frustration can indeed have a negative impact on mental health, it is essential to consider the potential role of other environmental and social factors in the development and perpetuation of MUPS [26]. For instance, socioeconomic factors, access to healthcare, and cultural influences may also contribute to the experience of unexplained symptoms. Economic disparities may restrict individuals' ability to seek timely medical care, while cultural beliefs may influence symptom perception and healthcare-seeking behaviors [27]. These factors collectively contribute to heightened psychological distress, impacting overall mental well-being and exacerbating the burden of unexplained symptoms [28].

Strengths and limitations

This pioneering study comprehensively investigates the association between MUPS and multiple domains including somatic symptoms, depression, anxiety, stress, and life events, filling a significant research gap within the Asian context. However, potential selection biases from purposive sampling and recall biases regarding stressors may limit generalizability. The confined geographic area and cross-sectional design with 200 patients hinder longitudinal insights. Establishing a cohort is essential for a deeper understanding. The study's implications extend beyond academia, emphasizing the need for holistic interventions and policy initiatives to address the impactful healthcare challenge posed by MUPS effectively.

## Conclusions

The study suggests an association between MUPS and psychiatric disorders, implying that MUPS may arise from or exacerbate psychological distress. Those with MUPS frequently experience heightened anxiety, depression, and other mental health challenges, illustrating the intricate link between somatic symptoms and emotional well-being. Additionally, our findings stress the importance of considering environmental and social factors, including gender and SES, to comprehensively understand MUPS.

## Appendices

We utilized three widely accepted and standardized assessment tools in our study, as illustrated in Table 6.

**Table 6:** Brief description of the assessment tools used in the study

Name of the tool	Description
Somatic Symptom Scale-8 (SSS-8)	The SSS-8 includes eight items that measure the severity and impact of somatic symptoms over the past seven days. The SSS-8 exhibits good reliability (Cronbach's α = 0.81), demonstrates high content validity from the PHQ-15, positive associations with depression and anxiety, and a stable factor structure across demographics, establishing it as a concise and valid tool for assessing somatic symptoms.
Depression Anxiety Stress Scale (DASS-21)	The Depression Anxiety Stress Scale (DASS-21) is a self-report questionnaire designed to measure levels of depression, anxiety, and stress. It demonstrates good reliability and validity, with Cronbach's α coefficients ranging from 0.88 to 0.94. The DASS-21 is widely used in research and clinical settings to assess negative emotional states accurately.
Presumptive Stressful Life Events Scale (PSLES)	The Presumptive Stressful Life Events Scale (PSLES) is a reliable measure designed to assess stressful life events experienced by individuals, adapted from the Holmes and Rahe Social Readjustment Rating Schedule. With 51 defined life events, it has been reconstructed and standardized for the Indian population, demonstrating high reliability (0.87) and validity in studies related to both mental and physical health.
